# Identification of *Histoplasma*-Specific Peptides in Human Urine

**DOI:** 10.1155/2012/621329

**Published:** 2012-03-26

**Authors:** David K. Crockett, Mark M. Kushnir, Joann L. Cloud, Edward R. Ashwood, Alan L. Rockwood

**Affiliations:** ARUP Institute for Clinical & Experimental Pathology, Department of Pathology, University of Utah, Salt Lake City, UT 84108, USA

## Abstract

Histoplasmosis is a severe dimorphic fungus infection, which is often difficult to diagnose due to similarity in symptoms to other diseases and lack of specific diagnostic tests. Urine samples from histoplasma-antigen-positive patients and appropriate controls were prepared using various sample preparation strategies including immunoenrichment, ultrafiltration, high-abundant protein depletion, deglycosylation, reverse-phase fractions, and digest using various enzymes. Samples were then analyzed by nanospray tandem mass spectrometry. Accurate mass TOF scans underwent molecular feature extraction and statistical analysis for unique disease makers, and acquired MS/MS data were searched against known human and histoplasma proteins. In human urine, some 52 peptides from 37 *Histoplasma* proteins were identified with high confidence. This is the first report of identification of a large number of *Histoplasma*-specific peptides from immunoassay-positive patient samples using tandem mass spectrometry and bioinformatics techniques. These findings may lead to novel diagnostic markers for histoplasmosis in human urine.

## 1. Introduction

Infection and disease caused by pathogenic and opportunistic fungi has been increasing over the past decade. One widespread and severe fungal disease, histoplasmosis, is caused by the dimorphic fungus *Histoplasma capsulatum*. The *H. capsulatum *mycelial phase may be inhaled by mammals to cause pulmonary disease [1]. Disease progression then occurs as the fungus transforms to yeast phase and disseminates to other parts of the body [2]. While most infections are mild, it can lead to rapid progression and life threatening symptoms for individuals with weak immune systems such as elderly, infants, HIV, and cancer patients or transplant recipients [3]. Furthermore, once infected, a latent infection may also be reactivated. Current laboratory testing for histoplasmosis does not provide adequate clinical sensitivity and specificity for accurate diagnosis of this infection. Antigen cross reactivity with other fungi species can be especially troublesome [4, 5]. 

Although progress has been made in understanding the pathogen-host interaction [6, 7], there are currently no known biomarkers of histoplasma infection, whereby molecular or chemical structures have been clearly defined. Incomplete genomes and/or databases for either protein or nonprotein compounds from *Histoplasma *have been a significant limitation of the work to date. This limitation has been improved due to recent efforts of the Human Microbiome Project hosted by the Genome Center at Washington University and the Broad Institute [8, 9]. The resulting genome and protein entries into public databases (such as NCBI) allow proteomics-based research to now move forward in a meaningful way. This study utilized comparative LC-MS/MS analysis of urine specimens from infected and noninfected patients to identify several potential protein and peptide markers specific to *H. capsulatum *infection in human urine.

## 2. Materials and Methods

Samples utilized in the study were unused aliquots of human urine samples referred for clinical laboratory testing at ARUP Laboratories (Salt Lake City, UT). Initial experiments included samples (*n* = 14) positive for histoplasma antigen (HAG) retrieved from samples submitted for routine clinical testing using an existing polyclonal enzyme immunoassay developed and validated by ARUP Laboratories using analyte-specific reagents (ImmunoMycologics, Inc., Norman, OK). Negative control samples (*n* = 14) were urine samples from individuals without histoplasma antigenuria. The study was approved by the Institutional Review Board of the University of Utah.

The following methodologies were used alone or in combination for sample preparation including immuno-enrichment (Immuno-Mycologics, Inc., Norman, OK), glyco-enrichment, SDS-PAGE gel separation, fractionation by molecular weight using ultrafiltration (Amicon Ultra-4, Millipore, Billerica, MA), high-abundant protein depletion (Agilent Technologies, Santa Clara, CA, Part #5185–5984) [10], deglycosylation (EDEGLY, Sigma-Aldrich, St. Louis, MO), reverse-phase fractions, and digestion using trypsin, glutamic C, or proteinase K (Princeton Separations, Adelphia, NJ). Experiments for discovery and validation of *Histoplasma* urine peptides were performed using nanoESI—tandem mass spectrometry (Agilent 6510 QTOF and AB/Sciex 4000 QTrap). Data was acquired (MassHunter 3.0 and Analyst 1.5) using replicate TOF scans as well as Auto MS/MS or information-dependent acquisition (IDA) MS/MS experiments. Replicate analysis was also performed using mass range partitions for precursor isolation of 400–600, 600–800, and 800–1600 m/z.

Accurate mass TOF scans were processed using MassHunter software (Agilent) for molecular feature extraction by retention time, mass-to-charge ratio, and integrated peak area. Files with molecular features (.mhd file format) corresponding to each analyzed sample were then uploaded into GeneSpringMS (Agilent) for further statistical analysis consisting of statistical filtering by normalized peak abundances, analysis of variance (ANOVA) and fold change (>2) between disease and control groups, significance testing, and unsupervised condition tree clustering to identify candidate features that were statistically significant and unique to the disease data set. Acquired MS/MS data files were searched using Mascot or Spectrum Mill (Agilent) against a combined human/histoplasma protein electronic fasta database (April 2011 NCBI download), and the results were evaluated.

Lastly, pooled samples in multiple independent experiments (*n* = 6) and additional individual samples (disease, *n* = 50 and control, *n* = 110) were used to validate potential biomarkers of HAG. Targeted MS/MS and extracted ion chromatograms (EIC) were also used to manually verify peptide targets. Potential nonhuman peptides identified in these experiments were further confirmed against the full NCBI nonredundant database using protein-protein BLAST (pBLAST).

## 3. Results and Discussion

**Figure 1:**
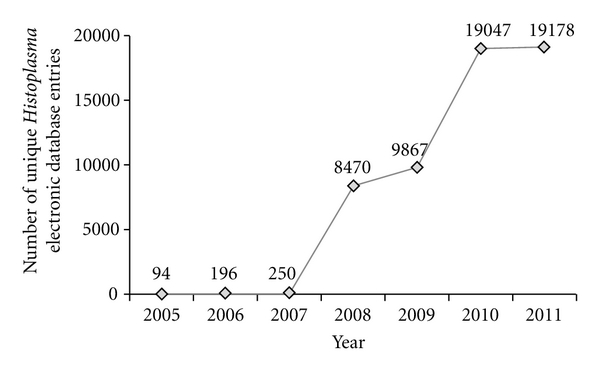
Rapid growth of electronic fasta database entries available for download at NCBI for *Histoplasma* proteins over last 7 years.

Although high-quality spectra were acquired during initial experiments, early search results were frustrated as electronic databases contained less than 250 protein entries for *Histoplasma*. Experiments during 2006 and 2007 identified only 5 potential peptides belonging to *Histoplasma*. Later experiments (2008-2009) yielded more promising results, but only an additional 14 peptides confidently identified. To better evaluate the newly added fungal sequences recently made public, all original data files (2006–2010) were combined and the MS/MS search repeated using the latest available NCBI electronic protein fasta entries (*n* = 19,178). Importantly, this increased number of protein entries led to an approximate 10-fold increase in the number of high-quality spectra matching *Histoplasma*-specific peptides. Further independent experiments were performed to confirm initial findings. This remarkable growth of electronic fasta database entries for *Histoplasma* proteins over the last 7 years, as available for download at NCBI is shown in Figure 1.

*Histoplasma*-specific peptides identified in this study are summarized in Table 1, where entries are ranked by the number of independent observations and BLAST score indicating “uniqueness” of the peptides. In total, 52 peptides were identified with a high degree of confidence, which belongs to 37 known and predicted *Histoplasma* proteins. Measuring one or more of these markers in patient samples may be potentially useful for diagnostic testing of histoplasmosis. 

## 4. Conclusions

Diagnosis and management of invasive fungal disease remains a major health problem, both in this country and worldwide. The ability to definitively diagnose histoplasmosis has become even more important due to the increasing number of patients with weakened immune systems who are susceptible to this life threatening disease, yet the laboratory tests for this disease are far from adequate.

Obviously, since fungal proteins and peptides are not expected to be present in urine from noninfected patients, studies similar to this represent a rich source of potential and novel targets for clinical diagnostic testing. The multiple and independent experiments with exhaustive sample preparation reported here have resulted in a unique data set representing the urine proteome associated with histoplasma antigenuria. Importantly, data files have been uploaded to the Proteome Commons Tranche repository (http://proteomecommons.org/) as a Histoplasma group project and are publically available as of May 2011.

## Figures and Tables

**Table 1:** *Histoplasma*-specific peptides identified from infected patient urine.

NCBI	Protein	Peptide	BLAST	ind.
accession	description	sequence	E-value	obs.^†^
154275270	polyubiquitin	LDVESADTIDAVK	0.006	5
		TLSDYNIQKESTLHLVLR	3*e*−08	4
		MQIFVK	1107	4
		IQDKEGIPPDQQRLIFAGK	2*e*−09	3
225554709	leucyl-tRNA synthetase	IDLLAVDK	141	4
		TVPSIFSSPWPDPLLSSR	2*e*−08	3
		NSSTISSPPR	3.8	3
240281880	conserved hypothetical protein	NPKSRPQHLQTATTNGR	4*e*−07	3
		SLEILRQR	78	3
240277865	DUF500 and SH3 domain-containing protein	NAEAAGAASTKGVAGVFSYSK	3*e*−09	3
		IGDRTGIFPSNYVDASNL	3*e*−08	3
240280678	5-oxoprolinase	GITGEYIHILVKPDMEK	5*e*−08	3
150412285	mannosyl-oligosaccharide 1,2-alpha- mannosidase	NHTSISVQDRDPPIR	9*e*−06	3
150408441	ADP-ribosylation factor	ITHSWVLQSLTRRAW	2*e*−06	3
239613150	predicted protein	KQTNYVSGLFRNSRT	2*e*−05	3
240276397	histone transcription regulator slm9	AISDLSWSPDGK	0.019	3
		SWSPDG	2675	3
150406597	hypothetical protein HCAG_00002	GWDQTGSNGANL	0.019	3
240273359	glycogen debranching enzyme	RLGLSALIRE	3.8	3
150408639	predicted protein	NLLTCMSR	24	3
240274939	ubiquitin-activating enzyme	NSFVNLALPFFSFIDPIASPMDK	1*e*−13	2
		LNNSEPR	535	3
240281152	conserved hypothetical protein	RQFVDSLSEGLGEQGKG	2*e*−06	3
239614048	TdiA	RGEHKYDPVVTLQPFGNKA	2*e*−9	2
240273135	xaa-proaminopeptidase	SKHQHFVGLSFDTISST	5*e*−07	2
		DIEFLIPIQCSILSERR	5*e*−08	2
		LDALRVPLTHLLAVR	5*e*−05	2
240276009	priB	RSKERSWLMLFVYDRS	1*e*−07	2
150411212	methionine sulfoxide reductase msrB	YFDSIPGAVTRKEDR	1*e*−05	2
239611175	predicted protein	TVIVITRRSLEPAR	3*e*−04	2
225555719	SAM and PH domain-containing protein	NWMRELMLARALK	1*e*−04	2
348156	YPS-3 yeast phase-specific protein	PAPFCGTCNPISGK	1e−04	2
150409767	predicted protein	KLDGAVPFKVQTRM	1*e*−04	2
239609148	phenol monooxygenase	MKPFTEEETIK	0.026	2
150407363	predicted protein	KCPTLSPENKN	0.084	2
154280483	predicted protein	LLFVGSNSAPGR	0.084	2
		TLPDDHILQEAK	0.011	2
150411980	conserved hypothetical protein	KLNMRVARWRE	0.011	2
225561657	conserved hypothetical protein	ISITDPANDR	1.2	2
239613737	cysteine synthase B	RVATSILRA	36	2
240275767	conserved hypothetical protein	LSIQATLIR	20	2
		LSIQA	75910	2
240276203	DNA binding protein URE-B1	AENTAESPESEDVKVK	9*e*−06	1
		ADPFATTPSKPR0	0.026	1
150414072	predicted protein	MFYFDSEFVGPPR	1*e*−04	1
		LLWGGAQQER	0.66	1
150412949	conserved hypothetical protein	RFSIKSCLFPHAKK	1*e*−04	1
150412547	predicted protein	RFLQPGDLVVLKS	0.003	1
150413459	predicted protein	KLSTLVGALATRN	0.019	1
240274117	pre-mRNA splicing factor	RVAEEMRCKV	0.27	1
154282923	2-isopropylmalate synthase	ACTLAAEPDR	1.6	1
239610650	glycerol:H+ symporter	KAVLASEPRT	5.2	1

^†^Number of times peptide(s) identified in independent experiments.

## References

[1] Durkin M, Kohler S, Schnizlein-Bick C (2001). Chronic infection and reactivation in a pulmonary challenge model of histoplasmosis. *The Journal of Infectious Diseases*.

[2] Chang MR, Taira CL, Paniago AMM, Taira DL, Cunha RV, Wanke B (2007). Study of 30 cases of histoplasmosis observed in Mato Grosso do Sul State, Brazil. *Revista do Instituto de Medicina Tropical de Sao Paulo*.

[3] Rachid A, Rezende LS, de Moura SF, Loffy PC, Magalhães FL (2003). A case study of disseminated histoplasmosis linked to common variable immunodeficiency. *The Brazilian Journal of Infectious Diseases*.

[4] Cloud JL, Bauman SK, Neary BP, Ludwig KG, Ashwood ER (2007). Performance characteristics of a polyclonal enzyme immunoassay for the quantitation of Histoplasma antigen in human urine samples. *American Journal of Clinical Pathology*.

[5] Xavier MO, Pasqualotto AC, Cardoso ICE, Severo LC (2009). Cross-reactivity of *Paracoccidioides brasiliensis*, *Histoplasma capsulatum*, and *Cryptococcus* species in the commercial platelia Aspergillus enzyme immunoassay. *Clinical and Vaccine Immunology*.

[6] Holbrook ED, Edwards JA, Youseff BH, Rappleye CA (2011). Definition of the extracellular proteome of pathogenic-phase *Histoplasma capsulatum*. *Journal of Proteome Research*.

[7] Winters MS, Chan Q, Caruso JA, Deepe GS (2010). Metallomic analysis of macrophages infected with *Histoplasma capsulatum* reveals a fundamental role for zinc in host defenses. *The Journal of Infectious Diseases*.

[8] Proctor LM (2011). The human microbiome project in 2011 and beyond. *Cell Host and Microbe*.

[9] Cuomo CA, Birren BW (2010). The fungal genome initiative and lessons learned from genome sequencing. *Methods in Enzymology*.

[10] Kushnir MM, Mrozinski P, Rockwood AL, Crockett DK (2009). A depletion strategy for improved detection of human proteins from urine. *Journal of Biomolecular Techniques*.

